# Mottled Mice and Non-Mammalian Models of Menkes Disease

**DOI:** 10.3389/fnmol.2015.00072

**Published:** 2015-12-18

**Authors:** Małgorzata Lenartowicz, Wojciech Krzeptowski, Paweł Lipiński, Paweł Grzmil, Rafał Starzyński, Olga Pierzchała, Lisbeth Birk Møller

**Affiliations:** ^1^Department of Genetics and Evolution, Institute of Zoology, Jagiellonian UniversityKraków, Poland; ^2^Department of Cell Biology and Imaging, Institute of Zoology, Jagiellonian UniversityKraków, Poland; ^3^Department of Molecular Biology, Institute of Genetics and Animal Breeding, Polish Academy of SciencesWólka Kosowska, Poland; ^4^Applied Human Molecular Genetics, Kennedy Center, Rigshospitalet, Copenhagen University HospitalGlostrup, Denmark

**Keywords:** Menkes disease, ATP7A, copper metabolism, mottled mice

## Abstract

Menkes disease is a multi-systemic copper metabolism disorder caused by mutations in the X-linked *ATP7A* gene and characterized by progressive neurodegeneration and severe connective tissue defects. The ATP7A protein is a copper (Cu)-transporting ATPase expressed in all tissues and plays a critical role in the maintenance of copper homeostasis in cells of the whole body. ATP7A participates in copper absorption in the small intestine and in copper transport to the central nervous system (CNS) across the blood-brain-barrier (BBB) and blood–cerebrospinal fluid barrier (BCSFB). Cu is essential for synaptogenesis and axonal development. In cells, ATP7A participates in the incorporation of copper into Cu-dependent enzymes during the course of its maturation in the secretory pathway. There is a high degree of homology (>80%) between the human *ATP7A* and murine *Atp7a* genes. Mice with mutations in the *Atp7a* gene, called *mottled* mutants, are well-established and excellent models of Menkes disease. *Mottled* mutants closely recapitulate the Menkes phenotype and are invaluable for studying Cu-metabolism. They provide useful models for exploring and testing new forms of therapy in Menkes disease. Recently, non-mammalian models of Menkes disease, *Drosophila melanogaster* and *Danio rerio* mutants were used in experiments which would be technically difficult to carry out in mammals.

## Introduction

Animal models are valuable tools for studying the role of copper (Cu) in various physiological, as well as pathological processes, such as neuro- and embryo development, inflammation and cancer (Lutsenko et al., 2007; Barry et al., 2010; Collins and Klevay, 2011). Over the last two decades, major progress has been made in our understanding of the molecular basis of Cu homeostasis, as well as in identifying the structure and function of Cu-containing proteins, largely thanks to animal models (Barry et al., 2010; Collins and Klevay, 2011; Telianidis et al., 2013).

## Mottled Mice as an Animal Model for Menkes Disease

Much of our knowledge originates from studies on mice with mutations in the *mottled* locus encoding the *Atp7a* gene (Kodama et al., 1993; Iwase et al., 1996; Kuo et al., 1997; La Fontaine et al., 1999; Niciu et al., 2007; Lenartowicz et al., 2010). Mouse models of *Atp7a* dysfunction are of particular importance for medical studies because mutations in the homolog human gene, *ATP7A* lead to the metabolic syndrome called Menkes disease, a fatal metabolic syndrome in humans (Menkes et al., 1962). The humane gene *ATP7A* and the murine gene *Atp7a* are both located on the X-chromosomes, and encode proteins called ATP7A, which belong to the P-type ATPase family. ATP7A transports Cu across membranes. During the catalytic cycle, ATP7A is autophosphorylated at a conserved aspartic acid and subsequently binds Cu to transmembrane domains. After releasing Cu, ATP7A is dephosporylated. ATP7A is located in the trans-Golgi network (TGN) in the absence of Cu, but recycles to the plasma membrane in the presence of Cu. It is assumed that *ATP7A* located in the TGN is involved in loading Cu on to Cu-requiring enzymes, whereas ATP7A located in the plasma is involved in extruding Cu from the cell. The two genes, *ATP7A* and *Atp7a* have a high degree of homology (>80%; Reed and Boyd, 1997; Kim and Petris, 2007).

The absence of a functional ATP7A transporter does not alter the Cu uptake, but greatly reduces the efflux of Cu ions to the extracellular environment (La Fontaine and Mercer, 2007; Lutsenko et al., 2007). ATP7A is the key mediator-protein involved in Cu transport across epithelial and endothelial cell barriers, and is therefore crucial for processes such as intestinal absorption and renal reabsorption of Cu and the ability of Cu to cross the blood-brain-barrier (BBB; Lutsenko et al., 2007). About 370 mutations have been identified in the *ATP7A* gene in humans, resulting in 3 different clinical phenotypes of Menkes disease: (1) Classical Menkes disease: patients with severe symptoms including mental retardation who die in early childhood (before the age of 6 years); (2) atypical Menkes disease: patients with less severe symptoms who live until adolescence; and (3) Occipital Horn Syndrome: patients with normal or near-normal neurological functions, milder symptoms, predominantly connective tissue problems, who live up to mid-adulthood. Furthermore mutations in ATP7A has also been associated with distal X-linked distal motor neuropathy (DMN; Kennerson et al., 2010) referred also as X-linked spinal muscular atrophy type 3 (SMAX-3; Yi and Kaler, 2014). Two different mutations leading to DMN has so far been identified (P1386S and T994I). Symptoms appeared when the patient was between 5 and 50 years of age and include gait instability with progressive weakness in feet and legs and loss of hand muscles. In contrast to Menkes disease DMN is not associated with symptoms of systemic copper deficiency.

**Figure 1:**
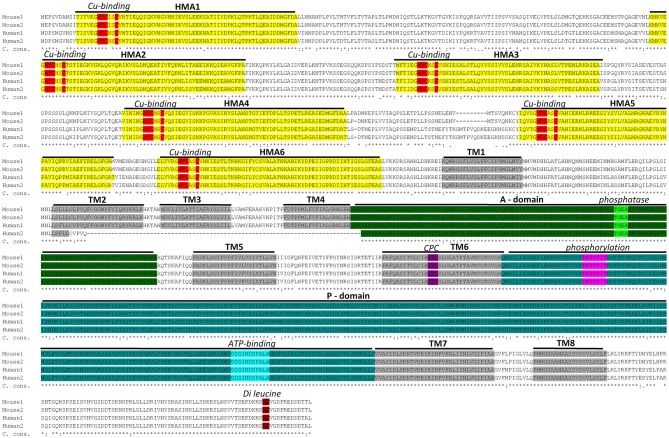
**Sequence alignment between mouse and human ATP7A proteins.** Two murine isoforms (NP_001103227.1 (Mouse 1) and NP_033856.3 (Mouse 2) and two human isoforms (NP_000043.4 (Human 1) and NP_001269153.1 (Human 2) are shown. Functional domains: HMA—heavy-metal-associated domain, TM—transmembrane domain, A-domain—actuator domain, P-domain—phosphorylation domain and corresponding functional motifs: Cu-binding, phosphatase, phosphorylation, ATP-binding and di-leucine are marked. It should be noted, that the human isoform 2 is shorter than isoform 1 and lacks a part of TM2 in addition to TM3 and TM4. This product is a result of alternative splicing, leading to skipping of exon 10. Isoform 2 is expressed at low level, in normal healthy individuals, but has been observed as the major product in a patient with a IVS10 mutation. Because the patient had OHS, in contrast to classic Menkes Disease, it has been suggested that isoform 2 has partly copper transporting activity (Qi and Byers, 1998). The sequence alignment is performed using Clustal Omega software (http://www.ebi.ac.uk/Tools/msa/clustalo/). Characteristic protein domains are marked based on conserved domains database (Marchler-Bauer et al., 2015) and previously published ATP7A protein structures (Kaler, 2011; Tümer, 2013). C. cons. – Clustal Omega consensus. An “*” (asterisk) indicates positions with fully conserved residues, a “:” (colon) indicates conservation between groups of strongly similar properties and a (period) indicates conservation between groups of weakly similar properties.

A sequence alignment between mouse and human ATP7A proteins is given in Figure 1. The amino acids sequences within functional domains are highly conserved between human and mouse ATP7A (Figure 1). Even between mouse, human, zebra fish and fruit fly ATP7A/ATP7 proteins, the functional domains are highly conserved, as demonstrated in supplementary Figure 1.

**Figure 2:**
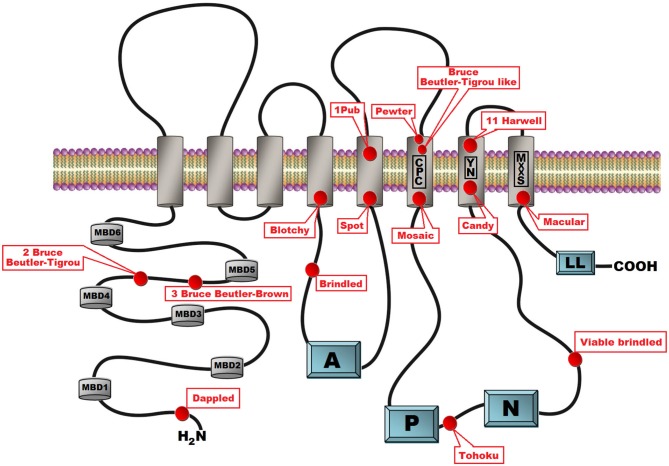
**Schematic presentation of the secondary structure of the ATP7A protein with location of the 15 mottled mutants indicated.** ATP7A is a transmembrane protein anchored to the membrane of the Golgi apparatus with eight transmembrane domains. The CPC amino acid motif within the 6th transmembrane domain is assumed to play a direct role in copper ions translocation across the biological membrane. The N-terminal peptide contains six cytoplasmic copper binding domains. Cytoplasmic domains are involved in the catalytic cycle that mediates cupric ions active transport at the cost of ATP hydrolysis. In the catalytic cycle ATP was bind to the nucleotide binding domains (N) and after hydrolysis the γ-phosphate of ATP is transferred to the invariant aspartate residue in the in the phosphorylation domain (P). Energy released by ATP hydrolysis is utilized for ions transport across a membrane. The actuator domain (A) located between the 4th and 5th transmembrane domains plays a key role in the dephosphorylation of the phosphorylated protein. The amino terminal part of the protein contains a dileucine motif (LL) motif that is involved in retrograde transport to the trans Golgi network (TGN).

Mice with mutations in the *Atp7a* gene are called *mottled* mutants reflecting the pattern of coat pigmentation in heterozygous females (Cecchi and Avner, 1996). Similarly to the large number of mutations in the human *ATP7A* gene, there are several murine strains with different mutations. The first description of mottled mutations in mice appeared from 1953 when Fraser and co-authors reported that “two mice with mottled coats have been analyzed genetically” (Fraser et al., 1953) and primary defects in copper transport in *mottled* mutants were described by Hunt (1974). Now, more than 60 years later and according to the Mouse Genome Informatics database, 109 *mottled* alleles are known and nearly half of them (*n* = 63) were introduced by gene trapping. Many *mottled* mutants have arisen spontaneously (*n* = 24) or have been induced by chemical (*n* = 7), chemical and radiation (4), radiation (*n* = 6), or targeted (*n* = 7) mutagenesis. Hemizygous *mottled* males exhibit a severe and often lethal phenotype. In general, affected males belong to one of three classes of phenotypic severity: (1) Mutant males that die *in utero*; (2) mutant males that die in the 3rd week of postnatal life; and (3) mutant males that die within a few postnatal months. So far, mutations in the *Atp7a* gene have been characterized in 15 *mottled* mutants (Table 1, Figure 2). The phenotypic diversity of the *mottled* alleles is a valuable source of knowledge not only about the molecular basis of Menkes disease, but also about the role of Cu in prenatal development.

**Table 1:** **Characteristic of the *mottled* mutations in mice**.

Allele symbol	Allele name	Exon/Mutation type	DNA change	Category	Mortality	References	Homologous mutations in human *ATP7A* gene
Atp7a^mo-blo^	Blotchy	11/ splice site mutation	c.2421 + 3A> C (IVS11 + 3A>G)	Spontaneous	Viable but reduced life span	La Fontaine et al. (1999)	c.2498 + 1G> A (Classic MD) Skjørringe et al. (2011)
Atp7a^mo-br^	Brindled	11/deletion	c.2396_2401-del6 p.(Leu799_Ala800del)	Spontaneous	Usually die when 2 weeks old	Grimes et al. (1997)
Atp7a^mo-2Btlr^	2 Bruce Beutler-Tigrou	5/nonsense	c.1492G> T (p.Glu498*)	Chemically induced	Die *in utero*	J:138687
Atp7a^mo-3Btlr^	3 Bruce Beutler-Brown	5/missense	c.1448T> C (p.Ile483Thr)	Chemically induced	Viable	J:153280
Atp7a^mo-^^Btlr^	Bruce Beutler-Tigrou like	15/missense	c.2993C> T (p.Ala998Val)	Chemically induced	Die *in utero*	J:133115	p.Ala1007Val (Møller et al., 2005) Mild
Atp7a^mo-^^pew^	Pewter	15/missense	c.2992G> A (p.Ala998Thr)	Spontaneous	Viable	Levinson et al. (1994)
Atp7a^mo-ca^	Candy	10/insertion	c.2153–2154ins81	Spontaneous	Die *in utero*	Cunliffe et al. (2001)
Atp7a^mo-dp^	Dappled	1/deletion	8990 bp deletion (2074 bp in promotor region, 104 bp in exon 1 and 6812 bp in intron 1) c.123?_c-19 + ?del)	Radiation induced	Die *in utero*	Haddad et al. (2014)	Several. Exact deletion not mapped Different phenotype (Tümer, 2013)
Atp7a^mo-11H^	11 Harwell	21/missense	c.4091C> A (p.Ala1364Asp)	Chemically and radiation induced	Die *in utero*	Kim and Petris (2007)	Ala1373Pro and Ala1373Val both classic MD (Gourdon et al., 2011)
Atp7a^mo-ml^	Macular	22/missense	c.4141T> C (p.Ser1381Pro)	Spontaneous	Usually die when 2 weeks old	Murata et al. (1997) Mori and Nishimura (1997)
Atp7a^mo-ms^	Mosaic	15/missense	c.2933G> T (p.Arg978Pro)	Spontaneous	Usually die when 2 weeks old	Lenartowicz et al. (2011)
Atp7a^mo-1pub^	1Pub	14/splice site mutation	c.2889 + 1G> A	Radiation induced	Die *in utero*	Cecchi et al. (1997)	c.2916 + 1G> A CS (Skjørringe et al., 2011)
Atp7a^mo-spot^	Spot	11–14/deletion	c.2380_2889del	Spontaneous	Die *in utero*	Cunliffe et al. (2001)
Atp7a^mo-tohm^	Tohoku	22i-23/deletion	c.4200-?_?del500?)p.Ile1061Ser) 1440-bp deletion between intron 22 and exon 23	Spontaneous	Die *in utero*	Mototani et al. (2006)
Atp7a^mo-vbr^	Viable brindled	16/missense	c.3107A> C p.(Lys1036Thr)	Spontaneous	Viable but reduced life span	Cecchi et al. (1997)

### Prenatal Lethal Mottled Mutants

Eight of the strains have mutations that lead to death *in utero* (class 1). This confirms that Cu plays an essential role in prenatal development, as well as the role of ATP7A in prenatal Cu metabolism. It has been suggested that mutations in the class 1 mice completely abolish the presence of functional ATP7A protein leading to the lethality of hemizygous males and the appearance of several pathological symptoms in heterozygous females.

Mutations in three strains lead to the absence of at least one exon in the *Atp7a* mRNA (Table 1). Fetuses of *dappled* (*Atp7a^mo−dp^*) hemizygous males with the genomic deletion of exon 1, are seemingly devoid of any *Atp7a* mRNA. Mutants display anatomical abnormalities and die already between day 17 and 18 of gestation (Silvers, 1979; Mercer et al., 1994). M*ottled spot* (*Atp7a^mo−spot^*) mutants have a genomic deletion including exons 11–14 (Cunliffe et al., 2001). A base pair substitution at the donor splice site of intron 14 leads to a constitutive Skipping of exon 14 in *1Pub* (*Atp7a^mo−pup^*; Cecchi and Avner, 1996). Male embryonic lethality at E11 of the tohoku mutants (*Atp7a^mo−tohm^*) is caused by a 1440-bp deletion between intron 22 and exon 23 of the Atp7a gene (Mototani et al., 2006).

Apart from deletions, vast insertions may also be responsible for lethality (Table 1). In c*andy* (*Atp7a^mo−ca^*), an insertion of an 81 bp sequence similar to the transposable element LINE in exon 10, leads to the activation of a cryptic splice site, resulting in a transcript that lacks the first 30 bp of exon 10 (Cunliffe et al., 2001). Not surprisingly, the nonsense mutation (c.1492G> T, p.Glu498*) in *Atp7a*^mo−2Btlr^ is prenatally lethal. Some missense mutations also lead to prenatal lethality in mice (Table 1). Miss-localization of ATP7A to the endoplasmic reticulum and impaired glycosylation of the protein have been shown in H11 (*Atp7a^mo11−H^*) mutants which were predicted to contain the amino acid substitution p.Ala1364Asp (Cunliffe et al., 2001; Kim and Petris, 2007). Also the missense mutation (c.2993C> T p.Ala998Val) leads to death *in utero* (REFJ: 133115).

There is also a group of less well characterized, predominantly chemically induced, prenatally lethal mottled mutations, which result in very small amounts of *Atp7a* transcript (Cecchi et al., 1997).

Interestingly, whereas wide deletions in the *Atp7a* gene in mouse mutant males have a prenatal lethal effect, in humans about 15% of the mutations in the *ATP7A* gene, which are caused by large deletions, in great majority result in the classical, severe form of Menkes disease leading to death in early childhood (<6 years; Poulsen et al., 2002; Tümer et al., 2003). It seems that even the most severe mutations in humans lead to a full-term pregnancy and live birth. This clearly indicates that the mutational effects on human *ATP7A* and mouse *Atp7a* genes can be different. These differences may be due to different levels of efficiency of placental Cu transport or by higher demands in mice for Cu supply during development.

### Mice Models for Classical Menkes Disease

Mutants belonging to the 2nd group are widely used for studies of Cu metabolism and serve as models of classical Menkes disease in humans. Especially *brindled*, *mosaic* and *macular* mice have been studied intensively. Although the genetic background of the three strains (*Atp7a^mo−br^*), (*Atp7a^mo−ms^*) and (*Atp7a^mo−ml^*) are different, their phenotypes are very similar (Table 1). In *brindled* mutants, the genetic defect is caused by the deletion of 6 bp in exon 11 which leads to an in-frame deletion of the amino acids Ala799 and Leu800 located in the region between the fourth transmembrane domain and the transduction domain of ATP7A protein (Grimes et al., 1997; La Fontaine et al., 1999). Although the ATP7A protein level in *brindled* cells is very similar to that of control cells, Cu transport activity of the mutant proteins is extremely reduced. In the *brindled* cells, ATP7A is located in TGN independently of the Cu level (note the normally Cu-dependent cellular trafficking; La Fontaine et al., 1999). In the *mosaic* mutants we identified the missense mutation c.2933G> C (p.Arg978Pro; Lenartowicz et al., 2012a; Table 1). Exon 15 encodes the highly conserved 6th transmembrane domain containing a CPC amino acid motif, which serves as a channel for Cu(I) transport and is critical for the protein function (Barry et al., 2010; van den Berghe and Klomp, 2010). In the *mosaic* mutants the ATP7A protein is mislocalized and is not translocated to the plasma membrane regardless of exposure of these cells to increased Cu concentrations (Lenartowicz et al., 2012a). Moreover, analysis of the coding region of the *Atp7a* gene in the *mosaic* mutants revealed the presence of the three additional nucleotides CAG (c.1417insCAG) at the end of exon 4, leading to the addition of a Glutamine between the 4th and 5th Cu-binding domain. This insertion was also associated with alternative splicing of the 3’ end of the exon 4 (Lenartowicz et al., 2004). However, this insertion was also found in other inbred strains of mice and is recognized as a polymorphism (Cecchi and Avner, 1996). Analysis of the coding region of the *Atp7a* gene in *macular* mutants revealed the mutation c.4223T> C (p.Ser1382Pro) in the region encoding the conserved 8th trans-membrane domain (Mori and Nishimura, 1997; Murata et al., 1997; Table 1). In the *macular* mutants, impaired Cu delivery to the secretory pathway is due to hyperphosphorylation of the ATP7A protein (Kim and Petris, 2007).

In mutants belonging to the 2nd group, the structure of the *Atp7a* gene is changed by missense mutations or small in-frame deletions, which lead to the synthesis of mutated protein with significantly reduced but not completely abolished activity. Consequently, hemizygous *brindled*, *mosaic* and *macular* males manifest pathological symptoms characteristic of strong Cu deficiency, but they survive until about the 3rd week of life. Actually, even few *brindled* and *mosaic* mutant males survive a critical period and can achieve remarkable longevity (Silvers, 1979; Lenartowicz et al., 2002; Kowal et al., 2010). *Mosaic* mutants that survive are relatively small and display defects in pigmentation, but do not develop neurological symptoms (Lenartowicz et al., 2002).

### Mottled Models for Occipital Horn Syndrome

Mutants of the 3rd group exhibit less severe phenotypes and survive to maturity. Mice in the 3rd group develop pathological symptoms similar to those described in patients with the atypical form of Menkes disease and with OHS. Patients with OHS have less profound neurological manifestations than those suffering from classical Menkes disease and their clinical features are mainly restricted to connective tissue defects. They also have a longer lifespan (Royce and Steinmann, 1990; Proud et al., 1996; Kaler, 1998; Godwin et al., 2006; Tang et al., 2006; Tümer and Møller, 2010).

Both *viable-brindled* and *blotchy* males manifest strong and numerous connective tissue abnormalities (Rowe et al., 1974; Das et al., 1995). The *viable-brindled* (*Atp7a^mo−vbr^*) mutants has the mutation c. 3107A> C in exon 16 (p.Lys1036Thr; Cecchi and Avner, 1996; Reed and Boyd, 1997). It results in constitutive post-Golgi localization of ATP7A and is probably caused by hyperphosphorylation of the mutated protein and significantly reduced activity (Kim and Petris, 2007). Nevertheless, v*iable-brindled* males suffer from aortic aneurysms, but they survive longer than mutants of the 2rd group, and succumb to blood vessel rupture at the age of 2–3 months (Rowe et al., 1974). V*iable-brindled* males are sterile.

The lifespan of *blotchy* males is the longest among *mottled* mutants. However these mutants suffer from emphysema, develop osteoarthrosis (Silberberg, 1977; Silvers, 1979), aortic and abdominal aneurysms due to defective elastin fibers (Silvers, 1979; Green, 1981). They usually die from a ruptured aorta at the age of more than 150 days (Rowe et al., 1974). Their average survival age is about 200 days and many live longer. Both *blotchy* males and females are usually infertile (Silvers, 1979). A splice-donor mutation is the cause of the defect in *blotchy* (*Atp7a^mo−blo^*) mice (Mercer et al., 1994; Das et al., 1995). The mutation (c.2421 + 3A> C) in intron 11 leads to partial skipping of 92 bp from exon 11 and results in a frame-shift (Mercer et al., 1994; Das et al., 1995; La Fontaine et al., 1999). *Blotchy* mutants produce, apart from inactive truncated forms, normal ATP7A protein, although in significantly reduced amounts. To explain the milder phenotype of the *blotchy* mutants it has been shown, that small amounts of the normal ATP7A protein are synthesized, which enables a limited Cu absorption and transport (La Fontaine et al., 1999). Similarly in humans, the less severe (atypical) form of Menkes disease and OHS are caused mainly by leaky splice-site mutations, permitting the production of small amounts of normal transcript (Møller et al., 2009; Skjørringe et al., 2011; Tümer, 2013). In humans, approximately 22% of mutations identified in *ATP7A* gene constitute splice-site mutations (Møller et al., 2009). However, splice-site mutations can also lead to the severe form of Menkes disease, if no normal transcript is produced (Skjørringe et al., 2011). In humans, an estimated 2–5% of wild-type transcript of the *ATP7A* gene, alleviates disease severity resulting in the OHS phenotype (Møller et al., 2000; Skjørringe et al., 2011).

The connective tissue abnormalities in the mutants are caused by decreased activity of lysyl oxidase which is responsible for connective tissue formation (Royce and Steinmann, 1990). Lysyl oxidase is a Cu-containing extracellular enzyme which catalyzes the process of oxidative deamination of lysine or hydroxylysine residues within the collagen α-chains, or of specific lysyl residues in elastin, to form the aldehyde precursors of the cross-links necessary to the formation of collagen and elastin (Royce and Steinmann, 1990; Rucker et al., 1998).

Significant reduction in lysyl oxidase activity has been demonstrated in skin and aorta (Royce and Steinmann, 1990) and in the medium of cultured fibroblasts obtained from OHS and Menkes patients (Kuivaniemi et al., 1985; Kemppainen et al., 1996).

It is interesting that *blotchy* mice have more pronounced connective tissue defects compared to *brindled* (2nd class mutant) despite *blotchy* mutants live much longer. Analysis of lysyl oxidase activity in extracts from the skin of 10-day old *brindled* and *blotchy* mutants revealed that the enzyme activity in *brindled* males was 50–60% of control level whereas it in *blotchy* males only was about 30% (Royce et al., 1982). This difference in lysyl oxidase activity may be explained by different molecular effect of the mutations. Whereas only small amount of wild type ATP7A is expressed in blotchy mutants as a result of a leaky splicesite mutation, normal amount of mutated ATP7A protein, trapped in TGN as described above, is expressed in *brindled* mutants (La Fontaine et al., 1999). The TGN trapped ATP7A protein probably allowed partial preservation of biosynthetic functions.

All *mottled* mutants exhibit defects in pigmentation caused by decreased activity of tyrosinase, a Cu-dependent enzyme that is involved in the formation of melanin pigment (Petris et al., 2000). The catalytic domain of apo-tyrosinase has two Cu-binding sites, and catalytic activity of tyrosinase requires the incorporation of Cu into both sites. This process is mediated by ATP7A (Petris et al., 2000). That is why the fur of the mutant males is uniformly pale, and the coat pigmentation in heterozygous females, which are mutation carriers, displays an irregular pattern, probably due to X chromosome inactivation (Lyon, 1962). Decreased tyrosinase activity has been found in *brindled* (Grüneberg, 1969; Mercer et al., 1999), *mosaic* (Styrna, 1977) and *macular* (Yamano et al., 1987) mutants.

### Mice Models for X-Linked Distal Motor Neuropathy

Conditional knockout mice with selective distruption of the *Atp7a* gene in motor neurons (*Atp7a^MN/Y^*) were used to explain the role of mutation in the *ATP7A* gene in development of DMN (Hodgkinson et al., 2015). Knockout mice have a normal lifespan and do not exhibit signs or symptoms of systemic copper deficiency characteristic for *mottled* mutants. They also have normal coat pigmentation. However during the lifespan knockout the mice developed gait defects, loss of limb strength, and muscle wasting, consistent with the disease progression observed in DMN patients (Hodgkinson et al., 2015).

## Lessons from the Mottled Mice

### Cu Uptake and Distribution

In agreement with the well established role of ATP7A as a transporter of Cu *via* the basolateral membrane of enterocytes (Lutsenko et al., 2007), the defective function of ATPase in *mottled* mice leads to the accumulation of Cu in the cytoplasm of the absorptive epithelial cells in the intestine (Camakaris et al., 1979; Shiraishi et al., 1988; Kodama, 1993; Nakagawa et al., 1993). Thus, results from studies in *macular* mice reveal a Cu accumulation in the cytoplasm of the intestinal epithelial cells and in the vascular endothelium (Kodama et al., 1993). This is consistent with the higher Cu level found in the small intestine of the *brindled* (Prohaska, 1983) and *mosaic* mutants (Lenartowicz and Sasuła, 2000). The molecular machinery involved in intestinal Cu transport and its regulation during early life in healthy mice is poorly understood.

In mice, the expression of the *Atp7a* gene begins early in prenatal life. *Atp7a* expression has been detected in only 7-day-old mouse embryos (Cecchi and Avner, 1996). It has been shown that its expression is ubiquitous during embryogenesis from day 9.5–18.5 (Kuo et al., 1997). Postnatally, *Atp7a* is expressed in all tissues and organs and *Atp7a* was categorized as a house-keeping gene (Linz and Lutsenko, 2007; Lutsenko et al., 2007). However, the expression level differs between organs and changes during the lifespan (Lenartowicz et al., 2011).

The pattern of Cu distribution in tissues from mouse models of Menkes disease cannot be classified as a general, systemic Cu deficiency. Several tissues such as kidney, intestine, placenta, and testis, have been shown to accumulate excessive or even toxic amounts of Cu (Prohaska, 1983, 1988; Phillips et al., 1986; Nakagawa et al., 1993; Lenartowicz and Sasuła, 2000; Kowal et al., 2010). This is very likely connected to the specific function of ATP7A in several cell types, which consists of expelling Cu from cells to the extracellular environment. Consequently, dysfunctional ATP7A in absorptive enterocytes and syncytiotrophoblasts does not only lead to the accumulation of Cu in these cells, but also limits this metal to other cells in the body. Furthermore, because ATP7A plays an imported roles in the delivery of Cu to Cu-containing enzymes, a large number of essential enzymes are left dysfunctional.

### Cu Delivery to the Foetus

During pregnancy, Cu is transported from the maternal to the foetal circulation via the placenta. This process is mediated by ATP7A and the homologous Cu-transporting ATPase, ATP7B (Hardman et al., 2007; La Fontaine and Mercer, 2007). It has been proposed that ATP7A plays a key role in Cu delivery to the foetus across the basolateral surface of the syncytiotrophoblast layer (Hardman et al., 2004, 2007), as an increase in the placental Cu content is observed in the *brindled* and *macular* mouse, and a direct blockage of placental transport has been demonstrated in these mouse models (Kasama and Tanaka, 1989; Xu et al., 1994).

Decreased transport of Cu across the placenta is probably the first reason for substantially reduced Cu content in the brain of 1-day-old *mosaic* mutant mice (Lenartowicz et al., 2011). Interestingly, wild-type mice and *mosaic* mutants show two different profiles of Cu content in the brain during early development (first 2 weeks after birth) i.e., a substantial increase is observed in wild-type mice, whereas no changes are found in mutants. This indicates that neonatal Cu deficiency in the brain of mutants just after birth is further aggravated throughout the early development (Lenartowicz et al., 2011).

### Cu Disturbance in the Brain

Menkes disease is commonly considered as a neurodegenerative, Cu deficiency disorder (Tümer and Møller, 2010; Kodama et al., 2012). In humans, Cu content in the brain (~5 μg/g tissue) is among the highest in various tissues (Collins and Klevay, 2011). This high Cu concentration is related to the metabolic activity of many Cu-dependent enzymes that function in the brain, such as cytochrome c oxidase, peptidylglicine alpha-amidating monooxygenase (PAM), dopamine-beta monooxygenase (DBH) and Cu, Zn-superoxide dismutase (SOD1; Suzuki and Gitlin, 1999; Prohaska and Broderius, 2006; Lutsenko et al., 2007; Nelson and Prohaska, 2009).

The *ATP7A* gene is highly expressed in the ependymal cells of the choroid plexus, a structure that regulates the concentration of the different molecules in the cerebro-spinal fluid, and the ATP7A protein is suggested to be involved in Cu transport across the BBB (Iwase et al., 1996; Niciu et al., 2006; Lutsenko et al., 2007). Copper deficiency in the central nervous system (CNS) of the Menkes patients lead to delay of their physical and mental development. In Menkes patients and *mottled* mutants, Cu accumulates in the cells comprising the blood-brain barrier and choroid plexus and is not transported from blood vessels to neuronal tissue (Kodama et al., 2005, 2012; Donsante et al., 2010, 2011). The severe decrease in Cu content in the brain and resulting deficiencies in the activity of Cu-containing enzymes are the main reasons for the early fatality in Menkes patients as well as in *mottled* mice.

ATP7A is especially abundant in the early postnatal period during brain development, because its activity is critical for proper axonal development and extension, and for synaptogenesis (El Meskini et al., 2005; Niciu et al., 2006, 2007). In the brain, ATP7A is expressed not only in the neurons, but also in a subset of astrocytes, microglia, oligodendrocytes, tanycytes and endothelial cells (Niciu et al., 2006, 2007). Neuropathological changes caused by mutations in the *Atp7a* gene are best characterized in *brindled* mutants. Due to the lack of activity of *Atp7a* in the brain of the *brindled* mutants the Cu content is 2–4 folds lower when compared to wild-genotype mice (Camakaris et al., 1979; Phillips et al., 1986). Detailed analysis of *Atp7a/ATP7A* expression patterns indicates that in the mutant’s brain, both transcript and protein levels are reduced in Purkinje cells, hippocampal pyramidal neurons and surrounding interneurons (Niciu et al., 2007). It resulted in the presence of numerous pycnotic neurons in the cerebral cortex and hippocampus in the *brindled* mutants (Niciu et al., 2007; Donsante et al., 2011). Electron microscopy examination of the Purkinje cells structure revealed cytoskeleton disruption, with tortuous, bulbous axons terminating abruptly in the cerebellar cortex (Niciu et al., 2007; Donsante et al., 2011). This explains why *brindled, mosaic* and *macular* mutant males in the 2nd week of life manifest severe neurological symptoms such as tremors, ataxia and seizures (Phillips et al., 1986; Kodama et al., 2005; Niciu et al., 2006; Lenartowicz et al., 2012a).

Interesting, neonates Menkes patients do not exhibit any neurological symptoms but patients older than 2 months developed seizures, prominent hypotonia and failure to thrive (Tümer and Møller, 2010; Kaler, 2011). Almost all Menkes patients older than 2 months developed epilepsy. Seizure types include focal or multifocal tonic-clonic, myoclonic, infantile spasms, and status epilepticus (White et al., 1993; Bahi-Buisson et al., 2006; Kaler et al., 2010). Electroencephalographic (EEG) analysis indicated that those patients exhibit abnormal electrocerebral activity (Kaler et al., 2008, 2010). Results obtained by Kaler et al. (2010) indicated that patients, who were presymptomatic diagnosed and early treated with copper injection (≤6 weeks of age), had decreased occurrence of seizures and improved brain electrical activity. In Menkes patients, magnetic resonance imaging (MRI) revealed neuropathological abnormalities including diffuse atrophy, ventriculomegaly, delayed myelination and tortuosity of cerebral blood vessels (Kaler et al., 2008; Kaler, 2011). Pathological changes are especially found in the cerebral cortex and the cerebellum, and include loss of Purkinje cells and neuronal loss of cerebellar molecular and internal granule cell layers (Kodama et al., 2012). In addition, subdural hematomas occurs secondary to abnormalities in brain arteries due to decreased activities of lysyl oxidase, which causes neurological damage (Kodama et al., 2012).

In the *mottled* mutants, neurological symptoms can also be caused by disturbances in neurotransmitter production. The Cu-dependent enzyme, DBH, catalyze the conversion of dopamine to noradrenaline which is metabolized to adrenaline. In the cells of patients with Menkes disease, the activity of DBH is significantly diminished and cannot be improved by parenteral Cu administration (Christodoulou et al., 1998). These findings indicate that Cu cannot be incorporated into the apo form of DBH in MD-affected cells because of ATP7A deficiency (Kodama et al., 2005). Reduced DBH activity is also found in the brain of *brindled* and *macular* mutants (Wenk and Suzuki, 1983; Kodama et al., 2005; Niciu et al., 2006; Bhadhprasit et al., 2012). The ratio of noradrenaline to dopamine and adrenaline to dopamine in these mutants is low when compared to wild-genotype controls (Kodama et al., 2005; Donsante et al., 2011, 2013; Bhadhprasit et al., 2012).

Another enzyme containing two Cu-centers which cycles between the Cu(I) and Cu(II) states during catalysis, is PAM (Steveson et al., 2003; Otoikhian et al., 2012). PAM catalyzes the C-terminal amidation of about 50% of all glycine-extended neuropeptides (Steveson et al., 2003; Bousquet-Moore et al., 2010). Such modification is essential for the bioactivity of numerous hormones and neuropeptides because it prevent COOH-terminus ionization and rendering it more hydrophobic. Such modification allow for better binding of α-amidated peptides to its receptors (Eipper et al., 1992, 1993; Bousquet-Moore et al., 2010). Lack of PAM activity in the PAM^−/−^ mice, lead to lethality during the prenatal life (Bousquet-Moore et al., 2010; Gaier et al., 2013b). Heterozygous PAM^+/−^ mice are fertile but developed pathological symptoms such as behavioral deficits, problems with thermoregulations and increased sensitivity to drug-induced seizures (Bousquet-Moore et al., 2010; Gaier et al., 2013b) and disturbance of copper metabolism in the brain (Gaier et al., 2013b). ATP7A co-locates with PAM in the intracellular compartments and is required for the maturation of this cuproprotein by metallation with Cu ions in TGN (Steveson et al., 2003; El Meskini et al., 2005; Otoikhian et al., 2012). In the brain of 12-day-old *brindled* mice, the expression level of PAM protein is similar to control mice, but the activity is reduced. In the *brindled* mutants it is manifested by reduced amidation of joining peptides, (JP-NH_2_), cholecystokinin (CCK) and a diminished level of alpha-melanotropin (alpha-MSH). Thus, the lack of amidated peptides in many target tissues, at crucial periods during the development, could contribute to the disease phenotype (Steveson et al., 2003; Niciu et al., 2007).

Epilepsy and seizures in Menkes patients and mottled mutants can be multifactorial processes connected with decreased copper contents in the CNS leading to deficits in neurotransmitter function, altered energy metabolism, and excitotoxicity (Prasad et al., 2011; Gaier et al., 2013a; Verrotti et al., 2014). It is known that some process of neural excitation and inhibition are modulated by amidated peptides (Bousquet-Moore et al., 2010). *In vitro* analysis of hippocampus slices indicated that the thyrotropin hormone-releasing hormone (TRP) reduce neuronal excitation by inhibition of potassium-stimulated glutamate and aspartate release. TRP analog administration reduce seizures (Nie et al., 2005; Veronesi et al., 2007; Bousquet-Moore et al., 2010). Results of immunostaining analysis indicated that both ATP7A and PAM highly expressed in the GABAergic neurons (Gaier et al., 2013b). Decreasing activity of PAM in the brain of the mottled mutants can lead to impaired production of amidated neuropeptide Y (NPY) and can be responsible for seizures. NPY decrease glutamate release and results obtained by Richichi and co-workers indicated that both acute and chronic seizures can be reduced by increasing NPY expression in the hippocampus (Richichi et al., 2004; Bousquet-Moore et al., 2010). *Macular* and *brindled* mutants also exhibit reduced cerebral cytochrom c oxidase activity (Donsante et al., 2011; Munakata et al., 2012).

The conditional knockout mice with selective disruption of *Atp7a* gene in motor neuron were very useful in evaluating the role of the ATP7A protein in normal motor neuron function. In (*Atp7a^MN/Y^*) knockout mice deletion of *Atp7a* resulted in disturbances of copper transport; copper concentration in the motor neurons increased with concomitant decreasing of Cu contents in the spinal cord. Accumulation of copper in the motor neurons lead to degeneration of the neuron cells and especially interesting degeneration process begins in the distal portions of axons and retreats towards the cell body (Hodgkinson et al., 2015). This pattern of neuron loss leads to denervation of the neuromuscular junctions and finally to muscular atrophy (Hodgkinson et al., 2015).

### Hepatic Cu

Examples from studies on iron absorption in rodents clearly show that the expression of the main apical and basolateral iron transporters in the duodenum is very low during the neonatal period (reviewed by Lipiński et al., 2013). Interestingly, in mice, the divalent metal transporter 1 (DMT1/Nramp2), is proposed in addition to functioning as an apical ferrous iron transporter (Lutsenko et al., 2007) to play a relevant role in physiological Cu absorption (Arredondo et al., 2003). DMT1 is barely detectable at postnatal days 0 and 5, but by day 10 this transporter is predominantly localized in the apical membrane of the maturing intestine (Lopez et al., 2006). Similarly to iron, the initial hepatic Cu stores established through maternal-fetal transfer, determine the iron status of the newborn.

Cu accumulated in the foetal liver during the second half of prenatal development is a primary source of this metal for meeting the needs of the organism during neonatal period (McArdle and Erlich, 1991; Mercer et al., 1992). Studies on *mosaic* mutants clearly show that the Cu content is significantly decreased already on day 1 after birth and persists at low level up to day 14 (Lenartowicz et al., 2011). An early decrease in hepatic Cu concentration has also been reported in other mouse models of Menkes disease (Prohaska, 1983; Phillips et al., 1986; Nakagawa et al., 1993). Considering that the liver is an organ that plays a role in the redistribution of Cu around the body, hepatic Cu scarcity in early development may result in restricted Cu supply from the liver to other tissues and organs including brain, heart or muscles. We recently reported the presence of low Cu levels in peripheral erythrocytes of 14-day-old *mosaic* male mice. This decrease strongly correlates with the down-regulation of both expression and activity of the Cu-dependent antioxidant enzyme SOD1, playing a crucial role in erythrocyte antioxidant defense. SOD1 dysfunction results in oxidative stress, increased fluidity of erythrocyte membranes and hemeolysis (Lenartowicz et al., 2014).

### Cu in the Kidney

A toxic Cu accumulation in the kidney is observed in most mouse models of Menkes disease (Suzuki-Kurasaki et al., 1997; Kirby et al., 1998; Lenartowicz et al., 2001, 2010). There is evidence that in *mottled* mice, renal epithelial cells of proximal tubules are the primary sites of Cu toxicity (Suzuki-Kurasaki et al., 1997; Kirby et al., 1998). Available data suggest a likely mechanism for this Cu overload. In the kidneys of healthy animals, Cu reabsorption from the urine occurs via the proximal renal tubules. The Cu ions are transferred back to the circulation via ATP7A, located in the basolateral membranes of epithelial cells of these tubules. This explains why the dysfunction of the ATP7A protein results in toxic Cu accumulation at the renal-proximal epithelium (Suzuki-Kurasaki et al., 1997; Kirby et al., 1998; Lenartowicz et al., 2010). Our previous results indicated that Cu accumulation in the kidney starts in prenatal life because the Cu-concentration in the only 1-day-old mice was higher than in wild-genotype mice (Lenartowicz et al., 2011). An increase in the Cu level in the kidney of the 18-day-old *macular* fetus was also found by others (Kasama and Tanaka, 1989). We also noticed that the Cu accumulation process in the kidney is strongly enhanced during the postnatal life of the mutants, because in 1-day-old *mosaic* males the Cu level was increased twofold, whereas it was increased fourfold in 14-day-old mutants when compared to control mice (Lenartowicz et al., 2010). It is noteworthy that Cu is even more active than iron in catalyzing the Fenton reaction. It is not surprising therefore that the kidneys of mouse *Atp7a* mutants display a large range of oxidative damage. Pathological changes involve renal tubules and glomeruli damage. In some cases the changes are very serious with renal tubules necrosis and sclerosis of renal glomeruli (Lenartowicz et al., 2002, 2010). Although renal pathology is not a direct cause of death in *mottled* mice, it constitutes a serious problem during Cu therapy. Parenteral supplementation with Cu prevents early death of mice, but at the same time it increases renal Cu content and strongly exacerbates kidney dysfunction (Lenartowicz et al., 2001, 2010). Treatment with Cu alone in postnatal life results in an up to 10-fold increase of Cu in the kidney of 14-day-old mutants when compared to untreated *mosaic* mice (Lenartowicz and Sasuła, 2000). The high Cu concentration persists during the life span of the treated mutants (Lenartowicz et al., 2010).

### Mottled Mice and Menkes Disease Therapy

Effective therapy is still a topical problem in Menkes disease. Treatment of Menkes patients with daily Cu injections may relieve the symptoms, as long as it is started within a few days after birth (Kaler et al., 2008; Tümer and Møller, 2010; Kodama et al., 2011, 2012). However, in the severe form of classic Menkes disease, even early Cu therapy is not always effective, and does not protect patients from neurological and connective tissue damage (Nadal and Baerlocher, 1988; Kaler et al., 1995; Choi and Zheng, 2009; Kodama et al., 2011, 2012).

*Mottled* mice appear to be a good model for exploring possible new therapeutic strategies of Cu administration in patients with Menkes disease. Cu-treatment applied as intraperitoneal or subcutaneous injections before the 7th day of postnatal life have been shown to improve the viability of *mosaic* (Kowal et al., 2010; Lenartowicz et al., 2010), *brindled* (Phillips et al., 1986) and *macular* mutant males (Shiraishi et al., 1988). Cu-treated *mosaic* mutant males survive significantly longer, achieve maturity and do not manifest neurological problems. Nevertheless, they still display a defect in pigmentation, and are usually smaller than control males (Lenartowicz et al., 2010). In both humans and mice, the BBB is immature during the first days after birth, and Cu can be pumped to the brain despite the lack of ATP7A activity. This is a critical period for initiating successful therapy (Kodama et al., 2011, 2012). In mice, if therapy starts later than day 10, almost all mutants die (Mercer et al., 1999).

Therapy in Menkes disease is mainly focused on Cu delivery to the CNS. A new and effective treatment consists of a “combined therapy”, in which Cu is administered together with lipophilic Cu chelators such as diethyldithiocarbamate (DEDTC) or dimethyldithiocarbamate (DMDTC). It is noteworthy that Cu in a lipid-soluble complex with DMDTC or DEDTC has a high capacity to penetrates the BBB (Tanaka et al., 1990; Kodama et al., 2005). Experiments performed on *macular* mice subjected to CuCl_2_ therapy on postnatal day 7 followed by combined CuCl_2_ and DMDTC therapy started at postnatal day 28, indicated that the combined treatment leads to a significant increase in the amount of Cu in the brain, and the treated mice survive longer than untreated ones (Tanaka et al., 1990; Kodama et al., 2005). We have also obtained very promising results by giving *mosaic* mice a CuCl_2_-DMDTC combined treatment prenatally starting on day 7 of pregnancy. *Mosaic* mutant offspring of females that underwent the combined CuCl_2_-DMDTC treatment show increased Cu level in the brain; this effect persists at least into the 2nd week of postnatal life. Furthermore, prenatal treatment with Cu compounds in *mosaic* mice lead to an increase in survival, improved locomotion performance, and a higher body mass (Lenartowicz et al., 2012b). Similar results were obtained in *macular* mice treated with Cu injections and oral disulfirm from postnatal day 7 (Bhadhprasit et al., 2012). Disulfirm is a dimer of DEDTC used for treating alcoholism and cocaine addiction (Bhadhprasit et al., 2012).

The activity of cytochrome c oxidase is higher in the brain of the Cu-DEDTC treated *macular* mutants when compared to the mutants treated with CuCl_2_ alone. Analysis of the catecholamine levels in the brain of the treated mice indicate that the dopamine levels are lower and the levels of noradrenaline and adrenaline are higher when compared to untreated mice, indicating that the DBH activity had improved with the treatment (Kodama et al., 2005). These findings suggest that DEDTC improves the transport of Cu to neurons in the brains of *mottled* mice making Cu available to Cu-dependent enzymes (Kodama et al., 2005).

Moreover, we and others have observed that the combined therapy efficiently reduces Cu accumulation in the kidney of treated *mottled* mice (Phillips et al., 1991; Tanaka et al., 1990; Kodama et al., 1993; Lenartowicz et al., 2012b), which might be a very important effect, considering the accumulation of Cu in the kidney of *mottled* mice and Menkes patients as a result of Cu treatment (Phillips et al., 1991; Kodama et al., 1993; Zaffanello et al., 2006; Kaler et al., 2008). Cu accumulation in the kidney induces a strong nephrotoxic effect (Lenartowicz et al., 2002, 2010).

Interestingly, reduced Cu accumulation in the kidney has also been achieved using the highly lipophilic organic Cu-complex, Cu-pyruvaldehyde bis(N^4^-methylthiosemicarbazone; Cu-PTSM). In the cells, Cu-PTSM is irreversibly reduced and cleaved to liberate Cu ions which can be incorporated into the intracellular Cu pools (Fujibayashi et al., 1993; Munakata et al., 2012). In *macular* mutants treated with Cu-PTSM, the renal Cu content was half of the Cu content in CuCl_2_-treated mutants. However there were no significant differences in brain Cu levels between CuCl_2_ and Cu-PTSM treated mutants, but cytochrome c oxidase activity was higher in Cu-PTSM treated *macular* mice (Munakata et al., 2012).

The next step in developing new effective treatments for Menkes disease was to study the possibilities of correcting the mutated *ATP7A* gene. *Brindled* mice served as an experimental model for the construction of transgenic mice that expressed the human *ATP7A* gene (Llanos et al., 2006; Donsante et al., 2011). Recombinant adeno-associated virus encoding a N-terminal truncated form of ATP7A (containing two instead of six copper binding sites) was injected into the lateral cerebral ventricles of neonatal *brindled* mutants. Transgenic *brindled* mice survived significantly longer than untreated mice and were fertile. However, the coat color and the Cu concentration in the organs of the rescued mutants resembled those in heterozygous females (Llanos et al., 2006). These results confirm the high degree of homology between the mouse and human *Atp7a/ATP7A* genes because the human gene was able to correct a genetic defect in mutant mice. Moreover, mutants treated with CuCl_2_ in combination with gene therapy survived even longer and the Cu level and activity of DBH in the brain was significantly increased, resulting in the improvement of neuromuscular strength and balance (Donsante et al., 2011).

## Interaction Between Cu and Iron: Lessons from Mouse Models of Menkes Diseases

The systemic iron homeostasis is maintained by the coordinated regulation of iron absorption in the duodenum, iron recycling of senescent erythrocytes in macrophages, and mobilization of iron stored in the liver. These processes are controlled by hepcidin, a key iron-regulatory hormone synthesized mainly in hepatocytes in response to iron stores, erythropoiesis, hypoxia and inflammation (Viatte and Vaulont, 2009). Hepcidin acts by posttranslational downregulating of ferroportin (Fpn), the only known iron exporter. The loss of Fpn decreases the iron flow into plasma from absorptive enterocytes, macrophages, and hepatocytes, thereby lowering plasma iron concentrations, contributing to intracellular iron arrest and thus leading to functional anemia. Apart from the degradation of Fpn triggered by hepcidin, multiple regulatory mechanisms control the amount of cell surface Fpn expression in tissue macrophages and absorptive enterocytes (Beaumont, 2010).

Some pathways of iron homeostasis largely depend on the activity of Cu-containing ferroxidases, such as ceruloplasmin (Cp) and hephastin (Heph). The close relationship between the biology of Cu and iron in mammals has been recognized and has for many years mainly been focused on the role of Cp expressed in macrophages, in facilitating iron recycling from senescent red blood cells (Collins et al., 2010). In humans, this process recovers approximately 25 mg iron *per* day, which corresponds to the daily requirement of iron for erythropoiesis. Cp is mainly synthesized in hepatocytes in the form of apo-Cp. Incorporation of Cu into apo-Cp results in the formation of the redox-active holoenzyme and is mediated by ATP7B during transit through the TGN. It has been shown that an alternative spliced glycosylphosphatidylinositol (GPI) variant of Cp (Cp-GPI) originally characterized in the brain (Patel and David, 1997) is of particular importance for iron metabolism (Collins et al., 2010; Prohaska, 2011). Cp-GPI cooperates with Fpn to facilitate the movement of iron out of cells. First, ferrous ions transported into the circulation by Fpn are oxidized by Cp-GPI and ferric ions are then bound by transferrin. Second, Cp-GPI is required for the stability of cell surface Fpn (De Domenico et al., 2007). The importance of Cp in human iron homeostasis has been confirmed by the description of the human congenital disease, aceruloplasminemia, in which mutations in the ceruloplasmin gene lead to its absence in plasma (Yoshida et al., 1995). While the anemia observed in humans with aceruloplasminemia is not severe, severe iron loading is found in the brain. Humans and mice suffering from aceruloplasminemia showed a striking impairment in the movement of iron out of reticuloendothelial cells and hepatocytes (Harris et al., 1995). It is not surprising therefore that iron-limited erythropoiesis is the main consequence of Cu deficiency. Metabolic pathways involving Cu and iron are also linked through the second Cu-containing ferroxidase Heph, which is implicated in iron efflux from the intestine (Collins et al., 2010). Heph co-localizes with Fpn on the basolateral membrane of duodenal enterocytes, where it stimulates the absorption of dietary iron. The *sla* (sex-linked anemia) mice have a defect in the basolateral export of iron from intestinal enterocytes into the circulation due to mutation in the hephastin gene, resulting in iron deficiency and microcytic hypochromic anemia (Vulpe et al., 1999). Systemic iron deficiency has also been reported in Cu-deficient mice that display decreased Heph ferroxidase activity in the intestine (Chen et al., 2006).

*Mottled* mice seem to be valuable for studying the interaction between Cu and iron metabolisms *in vivo*. Systemic iron homeostasis is mainly established and maintained by four cell types: intestinal enterocytes, reticuloendothelial macrophages, hepatocytes and progenitors of red blood cells. In this context, diverse tissue specific deregulated patterns of Cu content and distribution in *mottled* mice may increase our understanding of Cu-iron interactions at different sites in the body where iron balance is controlled. Recently, *brindled* mice served for the first time as an animal model in which iron homeostasis was studied (Gulec and Collins, 2013). Specifically, this study referred to the Cu-related compensatory response of the intestinal epithelium, the main site of iron entrance into the body in order to protect against iron deficiency. When *brindled* mice were deprived of iron, they developed anemia and enhanced intestinal iron absorption, similarly to wild-type mice. However, in contrast to controls, the upregulation of iron absorption in mutant mice was accompanied by increased enterocyte and hepatic Cu content as well as by increased serum ferroxidase (Cp) activity (Gulec and Collins, 2013). Additional mechanistic and molecular aspects of increased iron absorption in *Atp7a* mutants have been raised in rat intestinal epithelial cells (IEC-6), in which the *Atp7a* gene was silenced using short hairpin RNA technology. Transepithelial iron transport was increased in knockdown cells under both normal and iron-deficient conditions (Gulec and Collins, 2014). This phenomenon was associated with increased expressions of duodenal cytochrome b and Fpn involved in intestinal iron uptake and efflux, respectively (Gulec and Collins, 2014).

**Figure 3:**
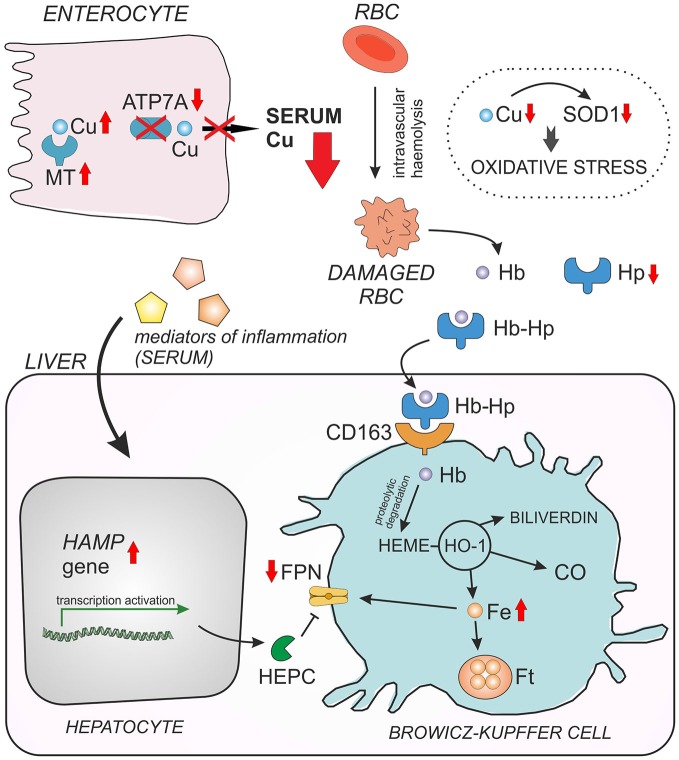
**Interaction between copper (Cu) and iron (Fe) in young *mosaic* mice.** Duodenal enterocytes can export copper across the basolateral membrane by ATP7A protein. Due to *ATP7A* gene mutation in *mosaic* mice, copper cannot be released to the serum and accumulates within the enterocytes in a complex with metallothionein (MT). Decreased serum Cu level entails Cu deficiency in red blood cells (RBC) and in consequence reduced activity/expression of Cu, Zn-superoxide dismutase (SOD1), which play a crucial role in RBC antioxidant defense. As the result, Cu-deficient RBC of *mosaic* mice display morphological abnormalities and undergo intravascular hemeolysis connected with hemoglobin (Hb) release to the serum and haptoglobin-dependent (Hp) elimination of free Hb from the circulation. When Hb is released from damaged RBC, it is instantly bound by haptoglobin (Hp) and forms a Hp–Hb high-affinity complex. This complex is then rapidly taken up from the circulation by the CD163 receptor present mainly on tissue macrophages (in the liver on Browicz-Kupffer cells). The CD163 receptor has no measurable affinity for free Hp. Thus, specific recognition of Hp–Hb by CD163 explains the decrease in Hp concentration in the serum during accelerated hemeolysis. The proteolytic Hb degradation in Browicz-Kupffer cells leads to the release of heme, which is then enzymatically decomposed by heme oxygenase 1 (HO-1) resulting in the formation of carbon monoxide (CO), biliverdin and Fe. Non-heme iron can be then stored as a complex with ferritin (Ft) or exported outside the cell by ferroportin (FPN), the sole cellular exporter of ionic iron known in mammalian cells. The content of hepatic non-heme Fe is elevated in *mosaic* mice, probably due to decreased expression of FPN. The concentration of cell surface Fpn largely depends on the post-translational regulation through internalization and degradation following hepcidin (Hepc) binding. Down-regulation of FPN expression in the liver of young *mosaic* mice is probably due to the concomitant up-regulation of hepatic hepcidin gene (*Hamp*), synthesized mainly in hepatocytes in response to systemic inflammation reported to occur in *mosaic* mice.

In our recent paper we used Cu-deficient *mosaic* mutants to investigate metabolic connections between Cu and iron (Lenartowicz et al., 2014). *Mosaic* mutant hemizygous males normally die by day 17 of life (Lenartowicz et al., 2012a) from severe Cu deficiency; therefore we used 14-day-old animals for our study. The erythrocytes of these mutants are Cu-deficient, display decreased activity/expression of the antioxidant enzyme SOD1, and have cell membrane abnormalities. SOD1 scavenges the superoxide anion, a reactive oxygen species contributing to the toxicity of iron via the Fenton reaction (Zelko et al., 2002). In consequence, the *mosaic* mice show evidence of hemolysis accompanied by haptoglobin-dependent elimination of hemoglobin (Hb) from the circulation, as well as the induction of heme oxygenase 1 (HO1) in the liver and kidney. Moreover, the hepcidin-Fpn regulatory axis is strongly affected in *mosaic* mice i.e., mutants show increased hepcidin and decreased Fpn expressions in the liver (Figure 3). Our results show for the first time the molecular mechanism of the induction of hemolysis by Cu deficiency and demonstrate how suckling mice adapt their iron metabolism to hemolytic insult (Lenartowicz et al., 2014).

## Other Animal Models of Menkes Disease

### *Danio rerio* and *Drosophila melanogaster* as Model Organisms of Menkes Disease

Although *mottled* mice have been a well-established and characterized mammalian model of Menkes disease for many years, recently scientists developed new non-mammalian models for this disorder such as the zebrafish (*Danio rerio*) and the fruit fly (*Drosophila melanogaster*) models. Both species have served as experimental animals in biology for many years due to relatively inexpensive maintenance and relative easiness for genetic manipulation. These new models expand our understanding of Cu metabolism in the cell and suggest new therapeutic strategies in Menkes disease.

Cu as a biometal plays a significant role not only in the adult organism, but it is also crucial in early developmental stages of humans and mice. It is difficult to follow the pathology of Cu metabolism during development in mammals. However, taking these facts into consideration, it seems that *ex vivo* fertilization, high fertility rate and rapid development make zebrafish an ideal model for such research. Embryos of *D. rerio* developed *ex utero* are optically clear, and therefore many of the processes of interest to the developmental biologist are easily observable. What is more, the genome of zebrafish has been sequenced and reveals that many genes and metabolic pathways are highly conserved between humans and zebrafish (Phillips and Westerfield, 2014).

*Danio rerio* absorbs Cu in two ways: via the intestine similarly to what is observed in mammals, and by the gills. Interestingly, the highest expression of the major Cu importer Ctr1 protein was detected in gut (Leung et al., 2014). Furthermore, the expression of *Atp7a* mRNA is ubiquitous in the zebrafish embryo and starts early in development (Mendelsohn et al., 2006). Besides it is believed that the Atp7a pump is necessary for loading maternal Cu into the egg (Madsen and Gitlin, 2008). Experiments have also shown that during embryogenesis Atp7a is responsible for proper Cu metabolism in individual cells. However Atp7a may not be essential for Cu transport from the yolk sac or within the embryo (Mendelsohn et al., 2006). In adult zebrafish, the expression pattern of *Atp7a* is similar to that found in mammalian tissues, such as intestine, kidney, heart, gills and liver (Chen and Chan, 2011; Leung et al., 2014). *Atp7a* is expressed most abundantly in the kidneys under elevated Cu level as well as under control conditions which suggests a major role of Atp7a in Cu export in *D. rerio* similar to observations in mice (Leung et al., 2014). However, in contrast to mice, in which Atp7a plays a significant role as a transporter in the brain barrier systems, glial cells and neurons (Lutsenko et al., 2010; Zheng and Monnot, 2012), the expression of A*tp7a* in the brain of *D. rerio* is very low (Chen and Chan, 2011). Surprisingly, in the liver of zebrafish the expression level of the *Atp7a* gene is high compared to the expression level of *Atp7b* (Leung et al., 2014), whereas the major Cu-ATPase in the adult mammalian liver is in fact *Atp7b* (Lutsenko et al., 2007). The *Atp7a/ATP7A* proteins in *D. rerio* and mammals play similar roles, and knockdown of the *Atp7a* gene (Mendelsohn et al., 2006; Madsen et al., 2008) as well as its inhibition by antisense oligonucleotide (Chen et al., 2011) result in a lack of pigmentation and the notochord deformation phenotype in embryos. These phenotypic effects are probably correlated to the dysfunction of Cu-dependent enzymes, i.e., tyrosinase and lysyl oxidase (Mendelsohn et al., 2006). These phenotypic effects are similar to what is observed in *mottled* mice (Kim and Petris, 2007) as well as in Menkes disease patients (Tümer and Møller, 2010). Moreover, expression of the human *ATP7A* gene in *Atp7a* zebrafish mutants can restore the normal phenotype (Mendelsohn et al., 2006). These data also confirm a high degree of conservation of Cu metabolism pathways between mammals and fish.

Since 2006, in Gitlin’s laboratory, three *calamity* mutants of zebrafish: *cal^vu69^* (Mendelsohn et al., 2006), *cal^gw246^* (Madsen et al., 2008) and *cal^gw71^* (Madsen and Gitlin, 2008), have been described as novel vertebrate models of Menkes disease. These strains were generated by N-ethyl-N-nitrosourea mutagenesis, and mutations in the *Atp7a* gene were confirmed by sequencing. Although these mutations are different, the phenotype effects are similar regarding the presence of notochord defects and the lack of pigmentation.

The mildest mutation, *cal^gw71^* results in a single, non-conservative amino acid substitution (p.Ile1061Ser) in the region close to the critical ATP binding residue (Glu1064) of the Atp7a protein. This single amino acid substitution results in significant depletion of functional protein in embryos. This *cal^gw71^* variant was, when expressed in fibroblasts obtained from patients with Menkes disease, able to deliver a suitable amount of Cu to the human Cu-dependent enzyme—tyrosinase. However, under Cu-depleted conditions, the activity of tyrosinase was reduced when compared to complementation with wild-type construct. Probably the mutated form of Atp7a retains some transport activity (Madsen and Gitlin, 2008). These data suggest that even low activity of Atp7a protein is sufficient to maintain an almost normal phenotype similarly to what we observed in humans (Møller et al., 2000; Skjørringe et al., 2011). Because the *cal^gw71^* animals are viable, it was possible to examine several post-embryonic effects of this mutation. Surprisingly, under adequate Cu conditions, adult homozygous mutants of zebrafish did not display a mutant phenotype, but in the absence of Cu, vertebral skeletal defects in larva were evident during development. What is more, treatment of larva with the chelator neocuproine (added to the water) leads to death, which indicates an increase in sensitivity to mild Cu deprivation as the embryo develops (Madsen and Gitlin, 2008).

Gitlin’s group has demonstrated the usefulness of morpholinos designed for targeting sequences near the mutant splice sites in order to force correct splicing and thereby correct aberrant splicing in an embryonic zebrafish (Madsen et al., 2008). Morpholinos which are oligonuclotides, usually 25 bases in length, are designed to target the RNA sequence of interest via complementary base pairing. The oligonuclotides bind to a target sequence thereby facilitating steric hindrance of proper transcript processing or translation (Bill et al., 2009). Injections of morpholino into *calamity* mutants fully rescue the Cu-deficient phenotype through the production of normal wild-type Atp7a. Morpholino oligonuclotides lead to a restoration of wild-type protein without detectable changes in the mRNA, suggesting a competitive translational regulation (Madsen et al., 2008).

*Drosophila melanogaster* has for several decades been one of the most powerful *in vivo* genetic model organisms used to describe molecular mechanisms of many biological pathways. What is more, the simplicity of its nervous system has made this small insect an especially valuable model in neurobiology. Thus, the fruit fly has contributed to the identification of genes and proteins underlying “molecular machinery” in the brain of processes, such as learning, memory formation, sleep or circadian rhythms. Moreover, since the nucleotide sequence of *D. melanogaster* genome has been published, it is presumed that approximately 10% of the genes common in both human and fly are involved in neurological diseases (Greenspan and Dierick, 2004). Therefore, *D. melanogaster* has also been used as a model for neurodegenerative diseases, like Huntington’s disease, Parkinson’s disease, Alzheimer’s disease and most recently Menkes disease.

Research into Cu metabolism in *Drosophila* began over 50 years ago. However, during the last decade scientists have particularly focused on the identification of regulatory mechanisms for cellular Cu metabolism. It has been proved that orthologs of the major eukaryotic Cu regulatory genes are well conserved between mammals and insects. Cu transporters and chaperons as well as a Cu-ATPase have been identified in the fruit fly (Norgate et al., 2007; Kirby et al., 2008; Hua et al., 2011). In contrast to mammals, *D. melanogaster* carries only one Cu-transporting ATPase known as *DmATP7*. The similarity between human ATP7A and ATP7B proteins is roughly 60%. Nevertheless, motifs responsible for basolateral targeting and retention of ATP7A are conserved in DmATP7, whereas targeting motifs in ATP7B are not (Southon et al., 2010). Similarly to human Cu-ATPases, DmATP7 is expressed from early embryogenesis. In adult *D. melanogaster* DmATP7 is expressed at high levels in the digestive tract and the nervous system (Norgate et al., 2006; Burke et al., 2008). DmATP7 seems to play the same role as the mammalian orthologs and is essential for Cu efflux in several *D. melanogaster* tissues (Southon et al., 2004; Burke et al., 2008). Furthermore, high conservation of Cu-ATPases between mammals and fruit fly permits the correction of the Cu hyper-accumulation phenotype of cultured fibroblasts from a Menkes disease patient carrying a null *ATP7A* mutation, by the expression of DmATP7 (Southon et al., 2010). In the fruit fly, the inhibition of DmATP7 expression in peptidergic neurons shows that neuropeptide amidation is perturbed (Sellami et al., 2012), and resemble that seen in the *brindled* mice, in which PAM is impaired (Steveson et al., 2003; Niciu et al., 2007).

Norgate et al. (2006) established a classical *D. melanogaster* model of Menkes disease with a null mutation. This mutant seems extremely lethargic, which could be explained by the dysfunction of the Cu-dependent nervous system or respiratory system. The mouth parts of the *DmATP7^−/−^* larva are smaller and reduced in pigmentation compared to controls. Moreover *DmATP7^−/−^* cell clones generated in control flies in the thorax and abdomen are depigmented. These observations indicate that DmATP7 supplies Cu to tyrosinase in *D. melanogaster* (Norgate et al., 2006) similarly to zebrafish (Mendelsohn et al., 2006; Madsen et al., 2008), mouse and humans (Petris et al., 2000). Unfortunately, *DmATP7^−/−^* insects are unable to grow after hatching and die early in development making it impossible to examine adult mutants (Norgate et al., 2006).

To overcome this problem, most recently Bahadorani et al. (2010) constructed a new *Drosophila* model of Menkes disease by conditional silencing of *DmATP7* only in the digestive tract. Expression of a RNAi construct against *DmATP7* was limited to gut cells. As is the case in Menkes patients and *mottled* mice, inhibiting the expression of *DmATP7* in the intestine enhanced lethality. It is hypothesized that the lethality of insects is the result of reduced Cu level in the nervous system. This hypothesis has been proposed on the basis of observations showing that the brains of the model flies were smaller and contained less stainable material when compared to control flies. Interestingly, roughly 50% of the flies survived to adulthood. However, in contrast to the *mottled* mice, such flies exhibit normal morphology without hypopigmentation or abnormalities of bristle hair-like structures. The surviving adults can be compared to OHS patients. The life span of such flies was only slightly shorter than that of control flies. Such adult survivors were sensitized to the oxidative stress of hyperoxia probably because of the reduced activity of SOD1 (Bahadorani et al., 2010). Reduction of SOD1 is also observed in the brain of Menkes disease patients (Shibata et al., 1995), mutant mice and *D. rerio* (Chen et al., 2011; Lenartowicz et al., 2014).

Studies on *Drosophila* Menkes disease model revealed MTF-1 as a potential therapeutic factor. The expression of MTF-1 in gut-*DmATP7-*RNAi larva enhances their survival to adulthood. A defensive mechanism probably involves the induction of methallothioneines, which bind excess Cu accumulated in the gut and prevent its toxic effect. The second scenario explains the therapeutic effect by the induction of the Cu importer (Ctr1B) expression, and thus the transfer of additional Cu to the tissues (Bahadorani et al., 2010).

## Concluding Remarks

To summarize, while murine models of Menkes disease are well-established, non-mammalian models can be used in experiments which are technically difficult to carry out in mammals. The rapid external development of the transparent zebrafish embryo permits a characterization of developmental abnormalities and allows cellular and genetic manipulations that are not possible during *in utero* development. The models can be used for large-scale screening for compounds that regulate Cu metabolism and could be potential drugs for Menkes disease treatment. The animals *D. rerio* and *D. melanogaster* are also invaluable to the development of new therapeutic strategies utilizing synthetic oligonucleotide analogs or the therapeutic effect of the MTF-1 protein.

## Author Contributions

ML, WK, PL and OP were drafting the work and substantial contributions to the conception and design of the work. PG, RS were revising manuscript critically for important intellectual content. LBM was revising manuscript critically for important intellectual content and design of the work.

## Funding

This work was supported by grant no. 2012/05/B/NZ4/02423 from the National Science Center of Poland.

## Conflict of Interest Statement

The authors declare that the research was conducted in the absence of any commercial or financial relationships that could be construed as a potential conflict of interest.
