# Supplementary material for: Mottled Mice and Non-Mammalian Models of Menkes Disease
Source: Front Mol Neurosci. 2015 Dec 18;8:72. doi: 10.3389/fnmol.2015.00072 (PMC4684000; doi:10.3389/fnmol.2015.00072)
Supplement: Supplementary file 1 [file Image_1.pdf]

```
                              Cu-binding                         HMA1
Mouse1          MEPSVDANSITITVEGMTCISCVRTIEQQIGKVNGVHHIKVSLEEKSATIIYDPKLQTPKTLQEAIDDMGFDALLHNANPLPVLTNTVFLTVTAPLTLPWDHIQSTLLKTKGVTGVKISP
Mouse2          MEPSVDANSITITVEGMTCISCVRTIEQQIGKVNGVHHIKVSLEEKSATIIYDPKLQTPKTLQEAIDDMGFDALLHNANPLPVLTNTVFLTVTAPLTLPWDHIQSTLLKTKGVTGVKISP
Human1          MDPSMGVNSVTISVEGMTCNSCVWTIEQQIGKVNGVHHIKVSLEEKNATIIYDPKLQTPKTLQEAIDDMGFDAVIHNPDPLPVLTDTLFLTVTASLTLPWDHIQSTLLKTKGVTDIKIYP
Human2          MDPSMGVNSVTISVEGMTCNSCVWTIEQQIGKVNGVHHIKVSLEEKNATIIYDPKLQTPKTLQEAIDDMGFDAVIHNPDPLPVLTDTLFLTVTASLTLPWDHIQSTLLKTKGVTDIKIYP
Danio           MALSTNLCRVTLGVEGMTCGSCVQSIEGRIGGLPGVIHIQVSLEQNNATVTYDHTQHTPQSIADAIEDMGFESSLTNATSTPVQTETKVFSKAGCSADSVQQALSTLAQIKGVIETQESA
Drosophila      ------------------------------------------------------------------------------------------------------------------------
All
All w/D.m.      *  *     :*: ****** *** :** :** : ** **:****::.**: ** . :**::: :**:****:: : *    ** *:* .:: :.  :   ::  *** : ***   :
All w/H2

                                                         Cu-binding                      HMA2
Mouse1          QQRSAVVTIIPSVVSASQIVELVPDLSLDMGTQEKKSGACEEHSTPQAGEVMLKMKVEGMTCHSCTSTIEGKVGKLQGVQRIKVSLDNQEATIVFQPHLITAEEIKKQIEAVGFPAFIKK
Mouse2          QQRSAVVTIIPSVVSASQIVELVPDLSLDMGTQEKKSGACEEHSTPQAGEVMLKMKVEGMTCHSCTSTIEGKVGKLQGVQRIKVSLDNQEATIVFQPHLITAEEIKKQIEAVGFPAFIKK
Human1          QKRTVAVTIIPSIVNANQIKELVPELSLDTGTLEKKSGACEDHSMAQAGEVVLKMKVEGMTCHSCTSTIEGKIGKLQGVQRIKVSLDNQEATIVYQPHLISVEEMKKQIEAMGFPAFVKK
Human2          QKRTVAVTIIPSIVNANQIKELVPELSLDTGTLEKKSGACEDHSMAQAGEVVLKMKVEGMTCHSCTSTIEGKIGKLQGVQRIKVSLDNQEATIVYQPHLISVEEMKKQIEAMGFPAFVKK
Danio           DNQGLAVTFVPSLVSEDQLGEVLKCLAPDTACRPPLSPKEGSTSRSFSGVEAVKMRIEGMVCLSCTTTIEGKIGKLKGVEKIKVSLESQEAAVVYLPYIITVDEIVKQIEVAGFKATVKS
Drosophila      ------------------------------------------------------------------------------------------------------------------------
All
All w/D.m.      :::  .**::**:*. .*: *::   *: * .     *    . *   :*   :**::***.* ***:*****:***:**::*****:.***::*: *::*:.:*: ****. ** * :*.
All w/H2

                                                Cu-binding                 HMA3
Mouse1          QPKYLKLGAIDVERLKNTPVKSSEGSQQKSPSYPSDSTTMFTIEGMHCKSCVSNIESALSTLQYVSSIVVSLENRSAIVKYNASLVTPEMLRKAIEAISPGQYRVSIASEVESTASSPSS
Mouse2          QPKYLKLGAIDVERLKNTPVKSSEGSQQKSPSYPSDSTTMFTIEGMHCKSCVSNIESALSTLQYVSSIVVSLENRSAIVKYNASLVTPEMLRKAIEAISPGQYRVSIASEVESTASSPSS
Human1          QPKYLKLGAIDVERLKNTPVKSSEGSQQRSPSYTNDSTATFIIDGMHCKSCVSNIESTLSALQYVSSIVVSLENRSAIVKYNASSVTPESLRKAIEAVSPGLYRVSITSEVESTSNSPSS
Human2          QPKYLKLGAIDVERLKNTPVKSSEGSQQRSPSYTNDSTATFIIDGMHCKSCVSNIESTLSALQYVSSIVVSLENRSAIVKYNASSVTPESLRKAIEAVSPGLYRVSITSEVESTSNSPSS
Danio           KPRQLKLSASEVERLLSAPKQTEEKL--SEPPADSTVTTLFQVTGMHCNSCVVNIQDNISKLPAVTSVVVSLENRQASIQHNPKQVSVVELQKAIEALPPGNFKAIIPASPE--------
Drosophila      -------------------------MPSDERVEATMSTVRLPIVGMTCQSCVRNITEHIGQKSGILGVRVILEENAGYFDYDPRQTDPARIASDIDDMG---FECSYPGDAAD-------
All                                            .     .   *. : : ** *:*** ** . :.    : .: * **:. . ..::   .    : . *: :    :.   ..
All w/D.m.      :*: ***.* :**** .:* ::.*      .*     .   *: * : ****:*** **:. :* *   *:*:*******.* :::* . *:    *:*****: ** ::. * :. *
All w/H2                                       .     .   *. : : ** *:*** ** . :.    : .: * **:. . ..::   .    : . *: :    :.   ..

                                    Cu-binding                 HMA4
Mouse1          SSLQKMPLNIVSQPLTQEAVININGMTCNSCVQSIEGVISKKPGVKSIHVSLANSTGTIEFDPLLTSPETLREAIEDMGFDAALPADMKEPLVVIAQPS--LE--TPLLPSSNELENV--
Mouse2          SSLQKMPLNIVSQPLTQEAVININGMTCNSCVQSIEGVISKKPGVKSIHVSLANSTGTIEFDPLLTSPETLREAIEDMGFDAALP-DMKEPLVVIAQPS--LE--TPLLPSSNELENV--
Human1          SSLQKIPLNVVSQPLTQETVINIDGMTCNSCVQSIEGVISKKPGVKSIRVSLANSNGTVEYDPLLTSPETLRGAIEDMGFDATLS-DTNEPLVVIAQPS--SE--MPLLTSTNEFYTKGM
Human2          SSLQKIPLNVVSQPLTQETVINIDGMTCNSCVQSIEGVISKKPGVKSIRVSLANSNGTVEYDPLLTSPETLRGAIEDMGFDATLS-DTNEPLVVIAQPS--SE--MPLLTSTNEFYTKGM
Danio           --------PGFLQPLVSVAEIHIEGMTCGSCVQSIEGTLSQKKGVRSAQVSLANHQGTFEYDPLLTSPEELRAAIEDMGFDAFLPETNSLVPSVVKSPS--PSVRSSSLSPVRSAVKENE
Drosophila      ---------PPETPASAWTNIRVVGMTCQSCVRNIEGNIGTKPGIHSIEVQLAAKNARVQYDPAQYDPAQIAELIDDMGFEASVQEPRSPSQSPSPAPASSPKKRATPTPPPP-SYAQNG
All                          *    : *.: **** ***:.*** :. * *::* .*.**   . .::**   .*  :    *:****:* :    .         *:   .
All w/D.m.                . ***.. : *.*:**** ********.:*:* **:* :*****   **.*:******** ** ********* *    .     *: .** .      *    ..  .
All w/H2                     *    : *.: **** ***:.*** :. * *::* .*.**   . .::**   .*  :    *:****:* :    .         *:   .

                                 Cu-binding          HMA5                                                      Cu-binding         HMA6
Mouse1          -------MTSVQNKCYIQVSGMTCASCVANIERNLRREEGIYSVLVALMAGKAEVRYNPAVIQPRVIAEFIRELGFGAMVMENAGEGNGILELVVRGMTCASCVHKIESTLTKHKGIFYC
Mouse2          -------MTSVQNKCYIQVSGMTCASCVANIERNLRREEGIYSVLVALMAGKAEVRYNPAVIQPRVIAEFIRELGFGAMVMENAGEGNGILELVVRGMTCASCVHKIESTLTKHKGIFYC
Human1          TPVQDKEEGKNSSKCYIQVTGMTCASCVANIERNLRREEGIYSILVALMAGKAEVRYNPAVIQPPMIAEFIRELGFGATVIENADEGDGVLELVVRGMTCASCVHKIESSLTKHRGILYC
Human2          TPVQDKEEGKNSSKCYIQVTGMTCASCVANIERNLRREEGIYSILVALMAGKAEVRYNPAVIQPPMIAEFIRELGFGATVIENADEGDGVLELVVRGMTCASCVHKIESSLTKHRGILYC
Danio           AESDAEPSTNTISKCFIQIGGMTCASCVANIERNLKNEYGIHSVLVALMASKAEVRYSPSVIDPLRIAELIRELGFTATVMDNYDGSDGSLELVVRGMTCASCVHKIESNLMKQKGILYA
Drosophila      SAVAIPVEQELLTKCFLHIRGMTCASCVAAIEKHCKKIYGLDSILVALLAAKAEVKFNANVVTAENIAKSITELGFPTELIDEPDNGEAEVELEIMGMTCASCVNKIESHVLKIRGVTTA
All                      .  .**:::: ********* **:. :.   *: *:****:*.****::.   *:     **: * **** : ::::   .:. :** : ********.**** : * :*:  .
All w/D.m.               .  .**:**: **************:.* **:*:******.******.*:**:*   ***:****** * *::*    .:* ******************.* *::**:*.
All w/H2                 .  .**:::: ********* **:. :.   *: *:****:*.****::.   *:     **: * **** : ::::   .:. :** : ********.**** : * :*:  .
```

```
                                                                            TM1
Mouse1SVALATNKAHIKYDPEIIGPRDIIHTIGSLGFEASLVKKDR--SANHLDHKREIKQWRGSFLVSLFFCIPVMGLMVYMMVMDHHLATLHHNQNMSNEEMINMHSAMFLERQILPGLSIMN
Mouse2SVALATNKAHIKYDPEIIGPRDIIHTIGSLGFEASLVKKDR--SANHLDHKREIKQWRGSFLVSLFFCIPVMGLMVYMMVMDHHLATLHHNQNMSNEEMINMHSAMFLERQILPGLSIMN
Human1SVALATNKAHIKYDPEIIGPRDIIHTIESLGFEASLVKKDR--SASHLDHKREIRQWRRSFLVSLFFCIPVMGLMIYMMVMDHHFATLHHNQNMSKEEMINLHSSMFLERQILPGLSVMN
Human2SVALATNKAHIKYDPEIIGPRDIIHTIESLGFEASLVKKDR--SASHLDHKREIRQWRRSFLVSLFFCIPVMGLMIYMMVMDHHFATLHHNQNMSKEEMINLHSSMFLERQILPGLSVMN
Danio           SVALSTNKAHIKYDPEVTGPRDIIRLIENMGFTASLVKKDR--PGSHLDHSREIRQWKRSFQISLFFCVPVMGMMIYMIVVDHMIDKYHQHHNATAEDRAKYHSTMFLEKQLLPGLSIMN
Drosophila      SVTLLTKRGKFRYITEETGPRSICEAIEALGFEAKLMTGRDKMAHNYLEHKEEIRKWRNAFLVSLIFGGPCMVAMIYFMLEMSDK------------G----HAN---MCCLVPGLSMEN
All             **:* *::.:::*  *  ***.* . *  :** *.*:.       .:*:*..**::*: :* :**:*  * *  *:*:::                     *:       ::****: *
All w/D.m.****:***********: ******: * .:** ********  ..****.***:**: ** :*****:****:*:**:*:** : . *:.:* : *:  : **:****:*:*****:**
All w/H2        **:* *::.:::*  *  ***.* . *  :** *.*:.       .:*:*..**::*: :* :**:*  * *  *:*:::                     *:       ::****: *
                            TM2                          TM3                                TM4
Mouse1          LLSLLLCLPVQFCGGWYFYIQAYKALKHKTANMDVLIVLATTIAFAYSLVILLVAMFERAKVNPITFFDTPPMLFVFIALGRWLEHIAKGKTSEALAKLISLQATEATIVTLNSENLLLS
Mouse2          LLSLLLCLPVQFCGGWYFYIQAYKALKHKTANMDVLIVLATTIAFAYSLVILLVAMFERAKVNPITFFDTPPMLFVFIALGRWLEHIAKGKTSEALAKLISLQATEATIVTLNSENLLLS
Human1          LLSFLLCVPVQFFGGWYFYIQAYKALKHKTANMDVLIVLATTIAFAYSLIILLVAMYERAKVNPITFFDTPPMLFVFIALGRWLEHIAKGKTSEALAKLISLQATEATIVTLDSDNILLS
Human2          LLSFLLCVPVQ------------------------------------------------------------------------------GKTSEALAKLISLQATEATIVTLDSDNILLS
Danio           LISFLFCVPVQFIGGRYFYCQAYKAVKHRTANMDVLIVLATTIAFTYSVVVLLVAMVERAKVNPITFFDTPPMLFVFISLGRWLEQIAKSKTSEALSKLMSLQATEATVVTLNEDMSVLS
Drosophila      LVMFLLSTPVQFFGGFHFYVQSYRAIKHGTTNMDVLISMVTTISYVYSVAVVIAAVLLEQNSSPLTFFDTPPMLLIFISLGRWLEHIAKGKTSEALSKLLSLKAADALLVEISPDFDIIS
All             *: :*:. ***                                                                             .******:**:**:*::* :* :. :  ::*
All w/D.m.*:*:*:*:***                                                                             .******:**:*******:***:.:  :**
All w/H2        *: :*:. **** ** :** *:*:*:** *:****** :.***::.**: :::.*:  . : .*:*********::**:******:***.******:**:**:*::* :* :. :  ::*
                                                     phosphatase                                                      TM5
Mouse1          EEQVDVELVQRGDIIKVVPGGKFPVDGRVIEGHSMVDESLITGEAMPVAKKPGSTVIAGSINQNGSLLIRATHVGADTTLSQIVKLVEEAQTSKAPIQQFADKLSGYFVPFIVLVSIVTL
Mouse2          EEQVDVELVQRGDIIKVVPGGKFPVDGRVIEGHSMVDESLITGEAMPVAKKPGSTVIAGSINQNGSLLIRATHVGADTTLSQIVKLVEEAQTSKAPIQQFADKLSGYFVPFIVLVSIVTL
Human1          EEQVDVELVQRGDIIKVVPGGKFPVDGRVIEGHSMVDESLITGEAMPVAKKPGSTVIAGSINQNGSLLICATHVGADTTLSQIVKLVEEAQTSKAPIQQFADKLSGYFVPFIVFVSIATL
Human2          EEQVDVELVQRGDIIKVVPGGKFPVDGRVIEGHSMVDESLITGEAMPVAKKPGSTVIAGSINQNGSLLICATHVGADTTLSQIVKLVEEAQTSKAPIQQFADKLSGYFVPFIVFVSIATL
Danio           EEQVDVELVQRGDVVKVVPGGKFPVDGRVIEGHSMADESLITGEAMPVTKKPGSTVIAGSINQNGSLLIKATHVGTDTTLSQIVKLVEEAQTSKAPIQQFADKISSYFVPFIVVISVLTL
Drosophila      EKVISVDYVQRGDILKVIPGAKVPVDGKVLYGHSSCDESLITGESMPVAKRKGSVVIGGSINQNGVLLVEATHTGENTTLAQIVRLVEEAQTSKAPIQQLADRIAGYFVPFVVVVSSITL
All             *: :.*: ****::**:**.*.****:*: ***  *******:***:*: **.**.****** **: ***.* :***:***:************:**::.****:*.:*  **
All w/D.m.************::*******************.**********:****************** *****:**************************:*.*******.:*: **
All w/H2        *: :.*: ****::**:**.*.****:*: ***  *******:***:*: **.**.****** **: ***.* :***:***:************:**::.****:*.:*  **
                                                               CPC          TM6                        phosphorylation
Mouse1LVWIIIGFQNFEIVETYFPGYNRSISRTETIIRFAFQASITVLCIACPCSLGLATPTAVMVGTGVGAQNGILIKGGEPLEMAHKVKVVVFDKTGTITHGTPVVNQVKVLVESNKISRNKI
Mouse2LVWIIIGFQNFEIVETYFPGYNRSISRTETIIRFAFQASITVLCIACPCSLGLATPTAVMVGTGVGAQNGILIKGGEPLEMAHKVKVVVFDKTGTITHGTPVVNQVKVLVESNKISRNKI
Human1LVWIVIGFLNFEIVETYFPGYNRSISRTETIIRFAFQASITVLCIACPCSLGLATPTAVMVGTGVGAQNGILIKGGEPLEMAHKVKVVVFDKTGTITHGTPVVNQVKVLTESNRISHHKI
Human2LVWIVIGFLNFEIVETYFPGYNRSISRTETIIRFAFQASITVLCIACPCSLGLATPTAVMVGTGVGAQNGILIKGGEPLEMAHKVKVVVFDKTGTITHGTPVVNQVKVLTESNRISHHKI
Danio           LAWIIIGFVNFSLVQTYFPGYDKSISEAEAVIRFAFQASITVLCIACPCSLGLATPTAVMVGTGVGAQNGILIKGGEPLEMAHKIQSVVFDKTGTITYGAPKVVQVKMLAEGNRLPRSKL
Drosophila      IAWIIIGFSNPNLVPVAMEH-KMHMDQNTIIVSYAFKCALSVLAIACPCALGLATPTAVMVATGTGAINGVLVKGATALENAHKVKTVVFDKTGTITHGTPMTSKVTLFVTAQVCSLARA
All             :.**:*** * .:* . :   .  :..   :: :**:.:::**.*****:***********.**.** **:*:**.  ** ***:: *********:*:* . :*.::. .:     :
All w/D.m.*.**:*** **.:*:******::***.:*::**************************************************:: *********:*:* * ***:*.*.*:: : *:
All w/H2        :.**:*** * .:* . :   .  :..   :: :**:.:::**.*****:***********.**.** **:*:**.  ** ***:: *********:*:* . :*.::. .:     :

Mouse1          LAIVGTAESNSEHPLGAAVTKYCKKELDT------ETLGTCTDFQVVPGCGISCKVTNIEGLLHKSNLKIEEN---NIKNA---SLVQ------IDAINEQSSTSSS-----------MI
Mouse2          LAIVGTAESNSEHPLGAAVTKYCKKELDT------ETLGTCTDFQVVPGCGISCKVTNIEGLLHKSNLKIEEN---NIKNA---SLVQ------IDAINEQSSTSSS-----------MI
Human1          LAIVGTAESNSEHPLGTAITKYCKQELDT------ETLGTCIDFQVVPGCGISCKVTNIEGLLHKNNWNIEDN---NIKNA---SLVQ------IDASNEQSSTSSS-----------MI
Human2          LAIVGTAESNSEHPLGTAITKYCKQELDT------ETLGTCIDFQVVPGCGISCKVTNIEGLLHKNNWNIEDN---NIKNA---SLVQ------IDASNEQSSTSSS-----------MI
Danio           LAIVGTAENSSEHPLGAAITKYCKQELGT------ESLGTCTDFQAVPGCGIRCLVSNTENLLKREDSDSEEN---Q-HNA---VLIQ------ISDARA-HSTEHP-----------LI
Drosophila      LTIVGAAEQNSEHPIASAIVHFAKDMLNVGATPQAGSFGKSSHFQAVPGCGIRVTVSNYEQTLRQACNADRIINYENLHRTHPQGSVPVDNGASIEHLLPQRSVRKSMELNNQQLLSDLV
All             *:***:**..****:.:*:.::.*. * .       ::*.. .**.******    *:* *  *::       .      : :.:      :        *.      *.               ::
All w/D.m.********..******:*:*****:** **:**** ***.****** * *:* * **::.: . *:*: :** *:**.  .    **.  :*
All w/H2        *:***:**..****:.:*:.::.*. * .       ::*.. .**.******    *:* *  *::       .      : :.:      :        *.      *.               ::
```

```
Mouse1         IDAH-----LSNAVNTQQYKVLIGNREWMIRNGLVISNDVDESMIEHERRGRTAVLVTIDDELCGLIAIADTVKPEAELAVHILKSMGLEVVLMTGDNSKTARSIASQVGITKVFAEVLP
Mouse2         IDAH-----LSNAVNTQQYKVLIGNREWMIRNGLVISNDVDESMIEHERRGRTAVLVTIDDELCGLIAIADTVKPEAELAVHILKSMGLEVVLMTGDNSKTARSIASQVGITKVFAEVLP
Human1         IDAQ-----ISNALNAQQYKVLIGNREWMIRNGLVINNDVNDFMTEHERKGRTAVLVAVDDELCGLIAIADTVKPEAELAIHILKSMGLEVVLMTGDNSKTARSIASQVGITKVFAEVLP
Human2         IDAQ-----ISNALNAQQYKVLIGNREWMIRNGLVINNDVNDFMTEHERKGRTAVLVAVDDELCGLIAIADTVKPEAELAIHILKSMGLEVVLMTGDNSKTARSIASQVGITKVFAEVLP
Danio          MDPQ-----PLTVVQTASYTVLIGNREWMRRNALQVRADVDEAMTEHERRGCTAVLVAVDNELCAMVAIADTVKPEAELAVHVLSAMGLEVVLMTGDNSKTARAIAAQVGIRKVFAEVLP
Drosophila     LEPEEELLTDQKIIDSPEILVLIGNREWMERNAIEVPLEISDCMTHEERKGHTAVLCALNGQLVCMFAVSDMVKPEAHLAVYTLKRMGIDVVLLTGDNKNTAASIAREVGIRTVYAEVLP
All            :: .        . ::: .  ********* **.: :  ::.: * ..**:* **** ::: :*  :.*::* *****.**:: *. **::***:****.:** :** :*** .*:*****
All w/D.m.:* : ..::: .*.********* **.* :  **:: * ****:* *****::*:***.::*************:*:*.:*****************:**:**** ********
All w/H2       :: .        . ::: .  ********* **.: :  ::.: * ..**:* **** ::: :*  :.*::* *****.**:: *. **::***:****.:** :** :*** .*:*****
                                    ATP-binding                                        TM7                                    TM8
Mouse1         SHKVAKVKQLQEEGKRVAMVGDGINDSPALAMANVGIAIGTGTDVAIEAADVVLIRNDLLDVVASIDLSRKTVKRIRINFVFALIYNLVGIPIAAGVFLPIGLVLQPWMGSAAMAASSVS
Mpuse2         SHKVAKVKQLQEEGKRVAMVGDGINDSPALAMANVGIAIGTGTDVAIEAADVVLIRNDLLDVVASIDLSRKTVKRIRINFVFALIYNLVGIPIAAGVFLPIGLVLQPWMGSAAMAASSVS
Human1         SHKVAKVKQLQEEGKRVAMVGDGINDSPALAMANVGIAIGTGTDVAIEAADVVLIRNDLLDVVASIDLSRKTVKRIRINFVFALIYNLVGIPIAAGVFMPIGLVLQPWMGSAAMAASSVS
Human2         SHKVAKVKQLQEEGKRVAMVGDGINDSPALAMANVGIAIGTGTDVAIEAADVVLIRNDLLDVVASIDLSRKTVKRIRINFVFALIYNLVGIPIAAGVFMPIGLVLQPWMGSAAMAASSVS
Danio          SHKVAKVEQLQQAGKRVAMVGDGVNDSPALAMADVGIAIGTGTDVAIEAADVVLIRNDLLDVVGSIDLSKKTVKRIRINFVFALMYNLVGIPIAAGVFMPVGLVLQPWMGSAAMALSSVS
Drosophila     SHKVAKIQRIQANGIRVAMVGDGVNDSPALAQADVGITIAAGTDVAAEASDIVLMRNDLLDVVACLDLSRCTVRRIRYNFFFASMYNLLGIPLASGLFAPYGFTLLPWMASVAMAASSVS
All            ******::::*  * ********:******* *:***:*.:***** **:*:**:********..:***: **:*** **.** :***:***:*:*:* * *:.* ***.*.*** ****
All w/D.m.*******:***: *********:*********:*****************************.*****:*************:*************:*:************* ****
All w/H2       ******::::*  * ********:******* *:***:*.:***** **:*:**:********..:***: **:*** **.** :***:***:*:*:* * *:.* ***.*.*** ****
                                                                                                                   di-leucin
Mouse1VVLSSLFLKLYRKPTYDNYELH-----PRSH---TGQRSPSEISVHVGIDDTSRNSPR------LGLLDRIVNYSRASINSLLSDKRSLNSVVT---SEPDKHSLLVGDFREDDDTTL---
Mouse2VVLSSLFLKLYRKPTYDNYELH-----PRSH---TGQRSPSEISVHVGIDDTSRNSPR------LGLLDRIVNYSRASINSLLSDKRSLNSVVT---SEPDKHSLLVGDFREDDDTTL---
Human1VVLSSLFLKLYRKPTYESYELP-----ARSQ---IGQKSPSEISVHVGIDDTSRNSPK------LGLLDRIVNYSRASINSLLSDKRSLNSVVT---SEPDKHSLLVGDFREDDDTAL---
Human2VVLSSLFLKLYRKPTYESYELP-----ARSQ---IGQKSPSEISVHVGIDDTSRNSPK------LGLLDRIVNYSRASINSLLSDKRSLNSVVT---SEPDKHSLLVGDFREDDDTAL---
Danio          VVLSSLLLKCYTKPTVEKLKRR-----LGDV---RTHGSLSDVSVHIGMGELRRPSPK------LSLLDRFVNYSRASINSLRSDKHSMNSMAL---SEPDKHSLLVGDDHCDNEIV----
Drosophila     VVCSSLLLKMYRKPTAKTLRTAEYEAQLAAERASGSEDELDKLSLHRGLDDLPEKGRMPFKRSSTSLISRIFMHDYGNITSP--DAKYGEGLLDPEEQYDGRTKIVRSRFHANDSTELQKL
All            ** ***:** * ***  .. .                  . . ..:*:* *: :  . .         .*:.*:. :. ..*.*   * :  :.:      .    : .:: .   : ::.
All w/D.m.******:** * ***  :. :     .   : * *::***:*: :  * **:*.****:*********** ***:*:**:. ************ : *:: .
All w/H2       ** ***:** * ***  .. .                  . . ..:*:* *: :  . .         .*:.*:. :. ..*.*   * :  :.:      .    : .:: .   : ::.
```

Supplementary figure 1. A sequence alignment of mouse, human, *Daniorerio* and *Drosophila melanogaster* ATP7A/ATP7 proteins. Conserved domains and motifs are marked with the same colors and abbreviations as in figure X. Sequence alignment was made with Clustal Omega software. Protein domains were marked based on conserved domains database (CDD, Marcher-Bauer at al. 2015) and previously published ATP7 and ATP7A protein structures (Norgate et al. 2006, Kaler 2011, Tumer 2012).Transmembrane domains of *Daniorerio* and *Drosophila melanogaster* ATP7A/ATP7 proteins were predicted using TMHMM Server v.2.0 (http://www.cbs.dtu.dk/services/TMHMM/). To better demonstrate the conservatism of the functional domains within regions which are missing in *Drosophila* ATP7 or human ATP7A isoform 2 additional alignments were performed without the particular sequence. All – Clustal consensus of all sequences, All w/D.m. – Clustal consensus of all sequences without *Drosophila* ATP7, All w/H2 – Clustal consensus without the sequence of human isoform 2. An * (asterisk) indicates positions with fully conserved residue, a : (colon) indicates conservation between groups of strongly similar properties and a . (period) indicates conservation between groups of weakly similar properties.
